# Genome-wide genetic overlap between fear-based disorders and generalised anxiety disorder

**DOI:** 10.1038/s41380-026-03677-2

**Published:** 2026-06-29

**Authors:** Abigail R. ter Kuile, Brittany L. Mitchell, Sang Hyuck Lee, Geneviève Morneau-Vaillancourt, Megan Skelton, Jonathan R. I. Coleman, Helena L. Davies, Jessica Mundy, Alicia J. Peel, Christopher Hübel, Molly R. Davies, Anna E. Fürtjes, Zain Ahmad, Yuhao Lin, Brett N. Adey, Thomas McGregor, Alish Palmos, Johan Zvrskovec, Matthew Hotopf, Gursharan Kalsi, Daniel J. Smith, David Veale, James T. R. Walters, Chérie Armour, Colette R. Hirsch, Andrew M. McIntosh, Naomi R. Wray, Sarah E. Medland, Enda M. Byrne, Nicholas G. Martin, Nathalie Kingston, John R. Bradley, NIHR BioResource, Nathalie Kingston, John R. Bradley, Hannah Stark, Carola Kanz, Alexei Moulton, Nigel Ovington, Jacinta Lee, Debbie Clapham-Riley, Katie Mills, Gerome Breen, Thalia C. Eley

**Affiliations:** 1 Institute of Psychiatry, Psychology and Neuroscience, King’s College London, London, UK; 2 National Institute for Health and Care Research Maudsley Biomedical Research Centre, South London and Maudsley NHS Foundation Trust, London, UK; 3 Department of Clinical, Educational, and Health Psychology, University College London, London, UK; 4 Brain and Mental Health program, QIMR Berghofer Medical Research Institute, Brisbane, QLD Australia; 5 School of Biomedical Sciences, Faculty of Medicine, The University of Queensland, Brisbane, QLD Australia; 6 National Centre for Register-based Research, Department of Public Health, Aarhus University, Aarhus, Denmark; 7 Clinic for Child and Adolescent Psychiatry, Psychotherapy and Psychosomatics, German Red Cross Hospitals Berlin Westend, Berlin, Germany; 8 School of Philosophy, Psychology and Language Sciences, The University of Edinburgh, Edinburgh, UK; 9 South London and Maudsley NHS Foundation Trust, Maudsley Hospital, London, UK; 10 Centre for Clinical Brain Sciences, University of Edinburgh, Edinburgh, UK; 11 South London and Maudsley NHS Foundation Trust, Bethlem Royal Hospital, Kent, UK; 12 Centre for Neuropsychiatric Genetics and Genomics, National Centre for Mental Health, Cardiff University, Cardiff, UK; 13 Research Centre for Stress, Trauma, and Related Conditions, School of Psychology, Queen’s University Belfast, Belfast, Northern Ireland UK; 14 Institute for Neuroscience and Cardiovascular Research, University of Edinburgh, Edinburgh, UK; 15 Institute for Molecular Bioscience, The University of Queensland, Brisbane, QLD Australia; 16 Queensland Brain Institute, The University of Queensland, Brisbane, QLD Australia; 17 School of Psychology, University of Queensland, Brisbane, QLD Australia; 18 School of Psychology and Counselling, Queensland University of Technology, Brisbane, QLD Australia; 19 Child Health Research Centre, The University of Queensland, Brisbane, QLD Australia; 20 National Institute for Health and Care Research (NIHR) BioResource, Cambridge Biomedical Campus, Cambridge University Hospitals, Cambridge, UK

**Keywords:** Genetics, Depression, Psychiatric disorders, Molecular biology

## Abstract

Twin studies reveal high genetic overlap between anxiety disorders and depression, contributing to the internalising spectrum. Some genetic specificity for fear-based anxiety disorders (fear), distinct from general anxiety and depression (distress), has also emerged. Limited datasets with detailed phenotyping across anxiety disorders have restricted most genome-wide association studies (GWAS) to “any anxiety diagnosis”. Additional genome-wide evidence to discern genetic differences between fear and distress is required. We conducted GWAS meta-analyses of fear (panic, agoraphobia, specific phobia, social anxiety disorder) and generalised anxiety disorder (GAD), measured using brief single-item and detailed symptom-based diagnoses from three datasets. We explored two control group criteria: phenotype-specific (fear/GAD) or broader anxiety/depression screening. We identified one independent genome-wide significant locus and three gene-level associations with fear (up to 35,523 N_cases_; 157,447 N_controls_). Four genome-wide significant loci and three gene-level associations were identified for GAD (up to 60,879 N_cases_; 117,064 N_controls_). The genetic correlation between fear and GAD was significantly different from unity only when excluding a depression-enriched dataset and using phenotype-specific control screening (r_g_ = 0.87; P = 9.32 × 10^−3^). Most complex traits had statistically similar genetic correlations with fear and GAD, including depression. Exceptions included general cognitive ability, educational attainment, and coronary artery disease, showing statistically stronger genetic correlations with fear than GAD, while bipolar disorder type I, anorexia nervosa, and neuroticism displayed the opposite pattern. Our findings partially support a distress-fear genetic distinction, but show stronger evidence for an overarching genetic liability to internalising psychopathology driving comorbidity across anxiety disorders and depression.

## Introduction

While high comorbidity exists across anxiety and depressive disorders [1–3], their distinct features form the basis of a model separating fear and distress [4, 5]. Fear-based anxiety disorders (panic disorder, agoraphobia, specific phobia, and social anxiety disorder) are characterised by context-specific fearful arousal and behavioural avoidance of a narrow range of stimuli [4, 5]. In contrast, generalised anxiety disorder (GAD) presents as uncontrollable worry about a broader range of situations. GAD is therefore often categorised alongside depression as a distress-based disorder, sharing core features of broad, pervasive negative emotions and exhibiting higher comorbidity with each other than with fear-based disorders [4–6]. Prior studies suggest an overarching shared liability alongside subtype-specific risk factors across distress-fear dimensions [5].

Twin studies estimate anxiety heritability in the range 20–60% [7–9], with evidence of genetic influences contributing to a broad internalising liability shared among individual anxiety disorders and depression [10–13]. However, other findings suggest this higher-order genetic liability can be differentiated into replicable lower-order genetic components. Fear-based disorders show partial genetic distinction from GAD, as a distress-based disorder, resulting in two distinct but correlated subfactors underlying anxiety genetics [10, 14].

While twin studies can examine the genetic structure of the anxiety disorder spectrum, molecular genetic approaches are needed to identify the specific genetic variants involved. Genome-wide association studies (GWAS) have begun identifying common genetic variants underpinning anxiety disorders, though fewer anxiety studies exist compared to other psychiatric disorders [15]. Recent large-scale GWAS have been conducted of broadly-defined anxiety and GAD symptoms [16–20], and one smaller panic disorder GWAS [21]. These studies revealed significant genetic correlations between anxiety and other psychiatric conditions, including depression, schizophrenia, bipolar disorder, and attention deficit hyperactivity disorder (ADHD) [16, 18–21]. Consistent with twin studies [10], preliminary genome-wide findings suggested that self-reported single-item diagnoses of any fear-based disorder (as a group) showed weaker genetic correlations with depression than between depression and GAD [22]. However, the common genetic variants associated with fear-based disorders remain underexplored, limiting our molecular-level understanding of the internalising genetic spectrum.

A previous panic disorder GWAS (~2250 cases) found a substantial genetic correlation with a combined ‘any anxiety disorder’ phenotype, although this did not survive multiple testing correction [21]. This and other fear-based disorder GWAS were underpowered for loci discovery or assessment of genetic correlations with a sufficiently broad range of traits. Understanding where fear disorders sit genetically in relation to other traits therefore remains limited [22–24]. Formal comparisons of fear and GAD genetic correlations with other psychiatric, behavioural, cognitive, and health traits could help identify transdiagnostic or subtype-specific mechanisms. Such analyses would strengthen molecular genetic evidence for hierarchical structures in the psychiatric spectrum, with the potential to improve diagnostic systems [5]. Further well-powered genome-wide evidence of the distress-fear genetic distinction is needed in the broader context of their relationships with other complex traits.

Anxiety disorder phenotyping is an important consideration when identifying genetic similarities and differences. Addressing comorbidity is a particular challenge in identifying anxiety-specific genetic influences, as anxiety disorders are among the most highly comorbid disorders across psychopathology [25]. Including heritable comorbid traits as GWAS covariates can bias results [26]. Screening controls for co-occurring traits in GWAS could inflate genetic correlations [27], necessitating the comparison of different screening approaches. Furthermore, genetic influences captured using brief phenotyping may be less specific to the focal trait than those from detailed phenotyping, reflecting more general psychopathology [28, 29]. Previous GWAS reporting distress-fear genetic correlations relied on brief, single-item reports of a diagnosis by a health professional for each fear disorder [22]. We previously showed that lower proportions of fear-based disorder diagnoses were reported via brief self-reports than when using detailed symptom-based measures [30]. The opposite pattern was observed for GAD [30]. Identifying genetic variants requires large sample sizes, creating a trade-off between maximising sample size and phenotypic detail. Combining detailed symptom-based diagnoses with brief single-item self-report diagnoses can achieve larger sample sizes [31], as demonstrated by a major depression GWAS that found strong genetic correlations across phenotyping methods [32].

In this study, we assessed shared and non-shared genetic influences on fear-based disorders and GAD at the level of common genetic variants. We increased sample size and power for loci-discovery and genetic correlation analyses by combining detailed symptom-based diagnoses with brief single-item diagnoses. We examined differences in genetic correlations between fear-based disorders and GAD with a range of other complex traits and assessed broad and specific approaches to screening controls. Our study represents significant progress in anxiety disorder genomics and provides further detail on the distress-fear distinction at the genome-wide level.

## Methods

### Datasets and measures

#### Overview

We conducted GWAS meta-analyses of two lifetime anxiety disorder phenotypes: (i) fear-based disorders ‘fear’; N cases = 35,523) and (ii) GAD (N cases = 60,879). Fear was defined as at least one of panic, agoraphobia, specific phobia or social anxiety disorder. We combined detailed symptom-based diagnoses and brief, self-report diagnoses from six samples, resulting in three case-control datasets (Table 1 & Supplementary Table 1).

**Table 1:** Sample sizes in each GWAS dataset and meta-analysis of fear-based disorders and GAD.

			Control criteria				N dataset total	
Anxiety disorder	GWAS Datasets	N_cases_	Anx-dep controls		Specific controls		(N_cases_ + N_controls_)	
			N_controls_	N_effective_	N_controls_	N_effective_	Anx-dep	Specific
**Fear**	GLAD+	18,091	4002	13,108	5,693	17,321	22,093	23,784
	UKB	10,789	83,236	38,204	137,209	40,010	94,025	147,998
	QIMR	6643	12,722	17,457	14,545	18,241	19,365	21,188
	‘*Restricted’* meta-analysis (GLAD+ & UKB)	28,880	87,238	51,312	142,902	57,331	**116,118**	**171,782**
	‘*Full’* meta-analysis (GLAD+, UKB & QIMR)	35,523	99,960	68,769	157,447	75,572	**135,483**	**192,970**
**GAD**	GLAD+	21,984	4002	13,543	6804	20,784	25,986	28,788
	UKB	26,067	83,236	79,402	95,715	81,950	109,303	121,782
	QIMR	12,828	12,722	25,550	14,545	27,265	25,550	27,373
	‘*Restricted’* meta-analysis (GLAD+ & UKB)	48,051	87,238	92,944	102,519	102,733	**135,289**	**150,570**
	‘*Full*’ meta-analysis (GLAD+, UKB & QIMR)	60,879	99,960	118,494	117,064	129,999	**160,839**	**177,943**

Numbers shown in bold are total sample size used in GWAS meta-analyses. Effective sample size was calculated as: N_effective_ = 4/ [(1/n_case_) + (1/n_control_)], which converts the power of a study sample size to one with a balanced case-control ratio (i.e. equivalent to a study with a sample prevalence of 50%, with N_cases_ = N_effective_ and N_controls_ = N_effective_).

#### GLAD+

The GLAD+ dataset included three samples. Cases were primarily from the Genetic Links to Anxiety and Depression (GLAD) Study, which recruited UK participants with a lifetime anxiety and/or depressive disorder [33]. Additional cases were from the United Kingdom Eating Disorders Genetics Initiative (EDGI UK), which recruits participants with a lifetime eating disorder [34], and the COVID-19 Psychiatric and Neurological Genetics (COPING) Study (Supplementary Materials) [35, 36]. The GLAD+ dataset used brief and detailed measures of all five anxiety disorders to define cases and controls. Controls were ascertained from the COPING Study [35, 36].

#### QIMR Berghofer

The Queensland Institute of Medical Research (QIMR) Berghofer dataset comprised cases from the Australian Genetics of Depression Study (AGDS) [37]. Controls were from the population-based QIMR Berghofer QSkin Sun & Health Study [38]. AGDS used detailed and brief measures, while QSkin used brief measures only. As all recruited AGDS participants had lifetime depression, this might increase depression/distress genetics in anxiety disorder GWAS, thus elevating the genetic correlation between fear, GAD, and depression. To address this potential depression-enrichment bias in anxiety cases, we conducted secondary ‘*restricted*’ meta-analyses excluding QIMR.

#### UK Biobank

The UK Biobank (UKB) is a population health cohort study [39]. Our dataset included a subset of UKB participants who completed the first Mental Health Questionnaire (MHQ1) [40]. The MHQ1 provided only brief measures for fear disorders and both brief and detailed measures for GAD (Supplementary Tables 1–2). We excluded GLAD+ participants who were also in UKB to prevent sample overlap.

#### Case-control definitions

We derived detailed symptom-based anxiety disorder measures using previously described algorithms [30] (Supplementary Table 3). Cases met DSM-5 criteria for a lifetime symptom-based disorder diagnosis, assessed using an online, self-report version of the Composite International Diagnostic Interview short-form (CIDI-SF) [37, 41]. Our brief measures comprised single-item self-report questions (e.g. “Have you ever been diagnosed with one or more of the following mental health problems by a professional?”; Supplementary Table 2). For panic assessments, we used the broad term “panic” to describe case-ness as we included both detailed diagnostic measures of panic disorder and brief measures of panic attacks. Although panic attacks are not specific to panic disorder, the brief diagnostic measure of panic attacks has shown agreement with detailed diagnostic measures of panic disorder [30].

We explored two control group definitions. First, we defined broadly screened controls as individuals without any anxiety disorder or depression (fear_anx-dep_ and GAD_anx-dep_). Due to the high genetic correlation between these disorders [22], this was the most powerful approach for loci discovery. Depression control screening was included as all AGDS cases had comorbid depression, with high comorbidity also in GLAD+ [3]. Second, we defined specifically screened controls (fear_specific_ and GAD_specific_) by excluding participants only for a diagnosis of the specific disorder being analysed. We did this to better distinguish genetic specificity between GAD and fear. Specific screening of controls was not possible in the QIMR dataset as we were limited to using a broad, single-item self-report diagnosis of “anxiety” (Supplementary Tables 1 and 2). Controls were not screened for the presence of other psychiatric disorders as this can bias genetic correlation estimates [27].

In summary, we conducted four GWAS meta-analyses with two control definitions (fear_anx-dep_, GAD_anx-dep_, fear_specific_ and GAD_specific_). We performed each GWAS meta-analysis in both the *‘full’* dataset (all three GWAS datasets) and the *‘restricted’* dataset (excluding the depression-enriched QIMR dataset). This resulted in eight meta-analyses in total. Within each meta-analysis dataset (‘full’ or ‘restricted’), cases for a given anxiety phenotype were identical between control definitions (i.e., fear_anx-dep_ vs fear_specific_); only the controls differed.

### Genome-wide association analyses

Genotyping and quality control were performed separately in each study (Supplementary Materials). Common genetic variants were imputed to the TOPMed imputation panel Version R2 on GRCh38 (GLAD+ and QIMR) or the Haplotype Reference Consortium and UK10K Consortium reference panels (UKB). Analyses were restricted to common variants (MAF > 1%) with imputation confidence INFO scores > 0.3 for TOPMed or > 0.4 for UKB.

We conducted four anxiety disorder GWASs: fear_specific_, GAD_specific_, fear_anx-dep_ and GAD_anx-dep_. Prior to meta-analysis, we performed a separate GWAS for these four phenotypes in each of our three datasets (GLAD+, QIMR, UKB ; Table 1). GWAS were conducted using REGENIE v3.1.3 (GLAD+, UKB) or SAIGE v0.44 (QIMR) [42, 43]. Covariates included the first ten principal components, genotyping batch, array (GLAD+ only), and assessment centre (UKB only). Analyses were limited to participants of European-associated genetic ancestry clusters, defined using principal component analysis (PCA). Prior to meta-analysis, GWAS summary statistics were processed and harmonised to the GRCh38 reference genome using the MungeSumstats R package (Supplementary Materials) [44].

### Meta-analyses and annotation

We used METAL to perform inverse-variance weighted meta-analyses of each anxiety disorder phenotype [45]. Primary meta-analyses (‘*full’)* included all three datasets, while secondary meta-analyses (‘*restricted’*) excluded QIMR to address potential depression-enrichment bias. We restricted common genetic variants to those overlapping across datasets in each meta-analysis. Meta-analysis results were mapped and annotated using FUMA v1.5.2 with default parameters applied and the “UKB release2b 10K European” reference panel [46]. MAGMA v1.08 with default parameters was used for gene-level and biological pathway analyses [47].

### SNP-based heritability

We estimated the liability-scale heritability captured by common genetic variants (h^2^_SNP_) of fear and GAD using LDSC regression [48]. We used the sum of effective sample size to adjust for variability in sample prevalence across datasets [49]. Population prevalences were derived from the COPING study, in which both detailed and brief measures were available to define cases and controls for all five anxiety disorders (Supplementary Table 4). Although not population-based, COPING study prevalence estimates aligned with epidemiological ranges for individual anxiety disorders [50, 51]. No epidemiological studies have estimated the population prevalence of fear-based disorders as a group. We therefore also calculated h^2^_SNP_ liability-scale estimates using prevalences 5% higher and lower than the COPING study prevalences.

### Global genetic correlations

Using LDSC regression [52], we calculated global genetic correlations (r_g_) between (i) fear and GAD and (ii) fear and GAD with 106 external complex traits. External traits spanned multiple domains, including psychiatric, cognitive/socioeconomic status, anthropometric, cardiovascular, behavioural, and physical health. Traits were considered sufficiently powered if they had an LDSC GWAS mean X^2^ > 1.02 and heritability Z score >4 [53]. We tested if fear-GAD r_g_ were significantly different from 0 and from 1. For differences from 0, we used default parameters in bivariate LDSC regression. For differences from 1, we used the chi-squared distribution function and [(|r_g_| −1)/se]^2^ in R. To correct for multiple testing, we applied a Bonferroni corrected *P*-value threshold (*α*/number of fear-GAD r_g_ tested; 0.05/4 = *P* ≤ 0.0125). Fear and GAD GWAS with an r_g_ significantly different from 1 were then tested for genetic correlations with external traits. We applied a Bonferroni-corrected significance threshold (*α*/number of external traits tested; 0.05/106 = *P* ≤ 4.71 × 10^−4^). For external traits with significant genetic correlations with GAD or fear, we tested if these correlations were statistically different between fear versus GAD using the block-jackknife LDSC method (Supplementary Materials) [52, 54]. Significant differences in fear-GAD r_g_ with external traits were determined using the P-value derived from the block-jackknife Z statistic. We applied a Bonferroni-corrected threshold adjustment for all external traits tested for r_g_ (*P* ≤ 4.71 × 10^−4^).

### Local genetic correlations

A global genetic correlation represents a genome-wide average and can mask heterogeneous effects at different regions and shared pleiotropic hotspots across traits [55]. We therefore conducted local genetic correlation analyses using LAVA (Local Analysis of [co]Variant Association) at 1226 genomic regions between fear-GAD, and with external traits showing differential fear-GAD global r_g_ (Supplementary Materials) [55]. Pairwise local r_g_ estimation was restricted to regions where both phenotypes showed local heritability (*P* ≤ 0.05). We applied false discovery rate (FDR) correction (*q* ≤ 0.1) across 1867 bivariate tests, as applied previously [56]. We assessed concordance between local and global r_g_ by calculating Pearson correlations between global LDSC estimates and average LAVA local r_g_ coefficients across (i) nominally significant (*P* ≤ 0.05) and (ii) FDR-significant regions.

Neuroticism follow-up analyses To dissect the differential fear-GAD global r_g_ with neuroticism total score, we conducted follow-up analyses of 14 neuroticism subcomponents: two genetic clusters (worry, depressed affect) and 12 individual items [57, 58]. We estimated bivariate fear-GAD global r_g_ using LDSC with block-jackknife testing for fear-GAD differences (Bonferroni correction: p ≤ 0.05/14 = 0.0036). To examine the genetic positioning of Fear and GAD alongside neuroticism subcomponents, we used multivariate genomic structural equation modelling (Genomic SEM) [59] (Supplementary Materials). We identified optimal factor structures encompassing r_g_ between fear, GAD and neuroticism subcomponents in two sets of analyses. The first included worry and depressed affect clusters plus four un-clustered items. The second included all 12 individual neuroticism items.

## Results

### Genome-wide association meta-analyses

**Fig. 1:**
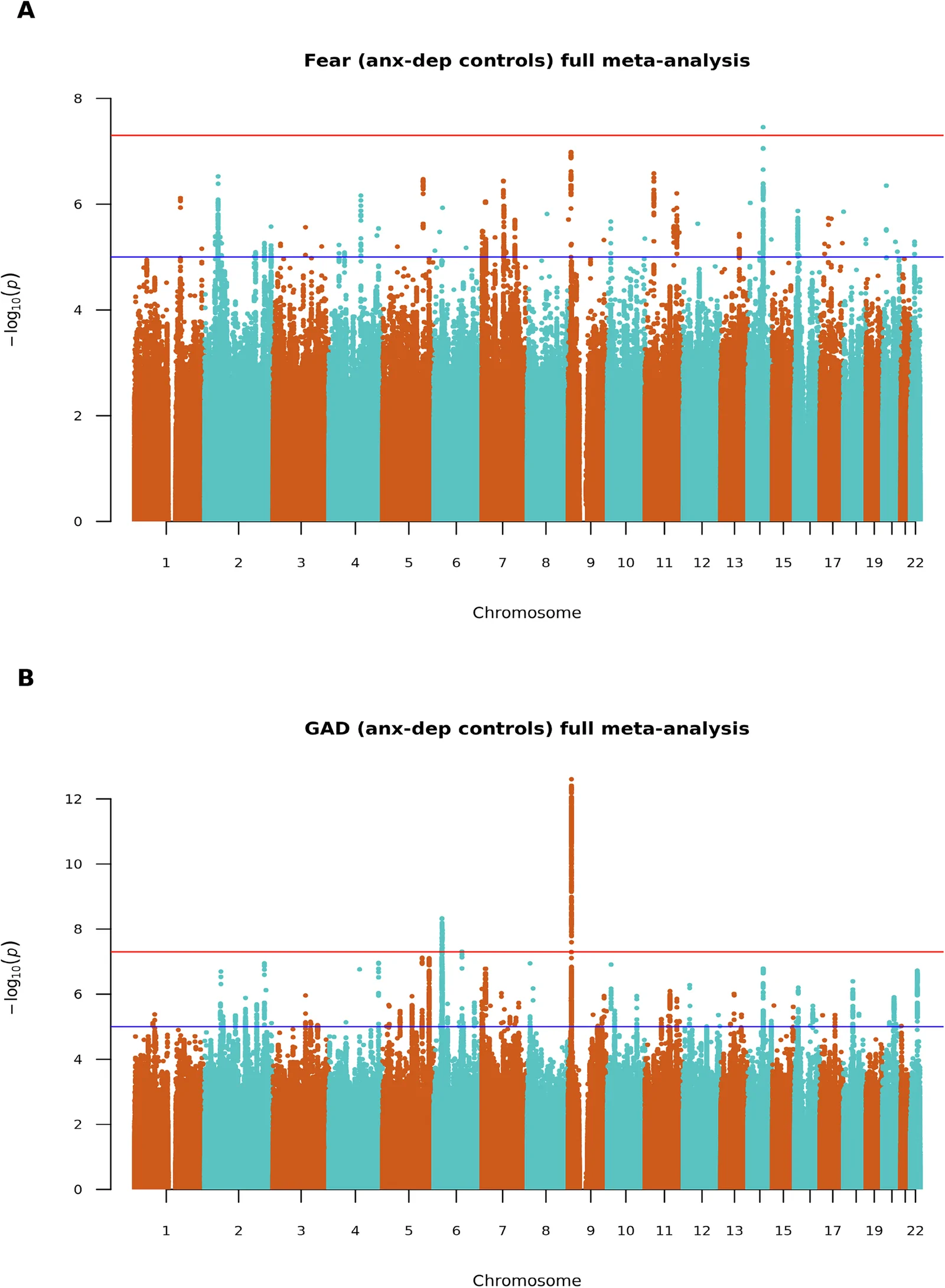
Genome-wide association study Manhattan plots for anxiety disorder phenotypes in the ‘*full*’ meta-analyses (GLAD+, UKB & QIMR datasets). Fear-based disorder GWAS **A** and generalised anxiety disorder GWAS **B** results from phenotypes with controls screened for any anxiety disorder and depression. Line red; common genetic variant genome-wide significance threshold (two-sided P ≤ 5 × 10^−8^), blue; suggestive significance threshold (two-sided P ≤ 1 × 10^−5^).

Manhattan plots for the most well-powered GWAS meta-analysis are shown in Fig. 1 (fear_anx-dep_ and GAD_anx-dep_ in the primary *full* meta-analysis; see Supplementary Fig. 1 for Q-Q plots). Manhattan plots and Q-Q plots for all other meta-analyses (fear_specific_, GAD_specific_ and the secondary *restricted* meta-analyses) are shown in Supplementary Figs. 1–4. We found little evidence of confounding in all GWAS meta-analyses (LDSC intercept = 1.00–1.01; Supplementary Table 5). LDSC intercepts >1 indicate some inflation, which is expected as the GWAS sample size increases [60]. The majority of inflation in our meta-analyses was due to polygenicity (93–100%), as indicated by LDSC attenuation ratio calculations (Supplementary Table 5). Regional plots for independent genome-wide significant loci are shown in Supplementary Figs. 5–9.

We identified one independent genome-wide significant locus (*P* ≤ 5 × 10^−8^) associated with fear in the *full* fear_anx-dep_ meta-analysis (lead variant rs10047892 on chromosome 14; *P* = 3.52 × 10^−8^; Fig. 1A; Table 2). No loci reached *P* ≤ 5 × 10^−8^ in the three other fear meta-analyses (fear_specific_ in *full*, or fear_specific_ and fear_anx-dep_ in *restricted*). Across our GAD meta-analyses, we identified four independent genome-wide significant loci associated with GAD. The most significant was on chromosome 9 in the GAD_anx-dep_ *full* meta-analysis (9p23; lead variant rs17189482; *P* = 2.49 × 10^−13^; Fig. 1B) and was genome-wide significant in all four GAD meta-analyses (Table 2). The locus on chromosome 6 (6p22.1) was the second most significant in GAD_anx-dep_ *full* meta-analysis (lead variant rs385816; *P* = 4.73 × 10^−9^), followed by locus 6q16.3 (lead variant rs17185536; *P* = 4.88 × 10^−8^; Fig. 1, **lower panel**). An additional locus on chromosome 2 (2p16.1) exceeded genome-wide significance in GAD_specific_ *full* meta-analysis (lead variant rs11688767; *P* = 3.53 × 10^−8^; Table 2).

**Table 2:** Independent genome-wide significant loci associated with anxiety disorder phenotypes.

Control screening criteria	Locus no.	Region	CHR	Lead SNP	Base pair	EA: NEA	Func; gene(kb)	P	OR	SE	N cand. SNPs	Ind. Sig. SNPs	Previous report in GWAS catalog
**Fear (full meta-analysis)**													
Anx-dep	1	14q24.3	14	rs10047892	75111346	T:C	intergenic; AREL1 (8793)	3.52E-08	1.07	0.01	41	rs10047892	EA, worry, systolic blood pressure
**GAD (full meta-analysis)**													
Anx-dep	2	9p23	9	rs17189482	11513617	G:T	intergenic; RP11-23D5.1 (237302)	2.49E-13	0.92	0.01	416	rs17189482 rs11515055	Neuroticism, depression, wellbeing, any anxiety disorder, anxiety symptoms, worry, BMI
	3	6p22.1	6	rs385816	29480224	A:G	intergenic; XXbac-BPG13B8.10 (1890)	4.73E-09	0.93	0.01	251	rs385816	SCZ, HDL, BIP, cognitive performance, depression, cardiometabolic & haematological traits, lung cancer, worry, smoking, neuroticism
	4	6q16.3	6	rs17185536	100620931	T:C	intergenic; RP3-344J20.1 (4153)	4.88E-08	0.95	0.01	5	rs17185536	LDL, weight, triglycerides, thyroxine levels, hip circumference, waist-hip ratio, sex hormone binding globulin
Specific	2	9p23	9	rs10960024	11616820	C:G	intergenic; RP11-23D5.1 (340505)	8.38E-13	0.93	0.01	426	rs10960024 rs11515055	As locus 2 above
	3	6p22.1	6	rs385816	29480224	A:G	intergenic; XXbac-BPG13B8.10 (1890)	2.86E-08	0.93	0.01	251	rs385816	As locus 3 above
	5	2p16.1	2	rs11688767	57988194	T:A	ncRNA intronic; CTD-2026C7.1 (0)	3.53E-08	1.05	0.01	24	rs11688767	Cross-disorder, sleep duration, SCZ, cognitive ability, depression, neuroticism, epilepsy
**GAD (restricted meta-analysis)**													
Anx-dep	2	9p23	9	rs17189482	11513617	G:T	intergenic; RP11-23D5.1 (237302)	9.31E-12	0.92	0.01	416	rs17189482 rs11515172	As locus 2 above
Specific	2	9p23	9	rs17189482	11513617	G:T	intergenic; RP11-23D5.1 (237302)	1.92E-11	0.92	0.01	416	rs17189482 rs11515172	As locus 2 above

Only Fear Anx-dep results shown; no loci reaching genome-wide significance (P ≤ 5 × 10 − 8) found in other fear meta-analyses. Locus no. = annotated locus number; CHR = chromosome; EA = effect allele; NEA = non effect allele; Func gene (kb) = functional consequence of the SNP on the nearest gene with distance in kilobase; OR = odds ratio; N cand. SNPs = Number of genome-wide candidate SNPs that are in LD (r2 = 0.6) with Ind. Sig. SNPs; Ind. Sig. SNPs = independent significant SNPs in the locus; Previous report = GWASs of other phenotypes that reported a genome-wide association with one or more of the candidate SNPs identified in this study (see Supplementary Tables 6 and 7 for fear and GAD loci results, respectively); *EA* educational attainment, *BMI* body mass index, *SCZ* schizophrenia, *HDL* high-density lipoprotein cholesterol, *BIP* bipolar disorder, *LDL* low-density lipoprotein cholesterol.

### Gene-level and gene-set association analyses

Three genes and two gene-sets were significantly associated with fear. *SNX29* (chromosome 16) was significantly associated with fear in all four meta-analyses. *PAX6* (chromosome 11) was significant in fear_anx-dep_ *full* meta-analysis, while *ERI3* (chromosome 1) was significant in fear_anx-dep_ *restricted* meta-analysis (Table 3). The gene-set suppression of apoptosis was associated with fear_specific_ and fear_anx-dep_ *full* meta-analyses (*P* = 7.89 × 10^−10^; Bonferroni correction threshold for 17008 gene-sets = *P* ≤ 2.94 × 10^−6^). Negative regulation of cell growth involved in cardiac muscle development was associated with fear_specific_ *full* meta-analysis (*P* = 3.32 × 10^−7^; Supplementary Table 8).

**Table 3:** Gene-level associations with anxiety disorder phenotypes.

Control screening criteria	Gene symbol	CHR	N SNPS	Z	P	P Bonf. Threshold (0.05/N genes)	Previous report in GWAS catalog	Gene name
**Fear (full meta-analysis)**								
Anx-dep	*SNX29*	16	2615	5.3	5.66E-08	2.68E-06 (18664 genes)	EA, insomnia, smoking, cognitive ability, depression, HDL, type 2 diabetes	Sorting nexin 29
	*PAX6*	11	42	4.69	1.38E-06		Neuroticism, depression, worry, blood pressure, BMI, insomnia, irritability, wellbeing	Paired box 6
Specific	*SNX29*	16	2616	5.27	6.94E-08		As above	As above
**Fear (restricted meta-analysis)**								
Anx-dep	*SNX29*	16	2618	5.41	3.22E-08	2.68E-6 (18675 genes)	As above	As above
	*ERI3*	1	192	4.69	1.34E-06		EA, alcohol consumption, height	ERI1 exoribonuclease family member 3
Specific	*SNX29*	16	2620	5.27	6.64E-08		As above	As above
**GAD (full meta-analysis)**								
Anx-dep	*TMEM106B*	7	195	4.78	8.84E-07	2.68E-6 (18665 genes)	Anxiety, depression, EA, wellbeing, insomnia, Alzheimer’s, neuroticism, GAD symptoms, PTSD, HDL	Transmembrane protein 106B
	YLPM1	14	155	4.67	1.47E-06		Anxiety, EA, neuroticism, blood pressure, HDL, body fat %, cortical thickness	YLP motif-containing protein 1
	*SORCS3*	10	1818	4.6	2.16E-06		Anxiety, EA, depression, blood pressure, externalising behaviours, CAD, insomnia, neuroticism, smoking, ADHD	Sortilin related VPS10 domain containing receptor 3

Gene-level association analyses conducted in MAGMA. Only analyses exceeding Bonferroni-corrected significance thresholds shown. CHR = chromosome; Previous report = GWASs of other phenotypes that report genome-wide significant loci that map onto the gene-level associations identified in this study (see Supplementary Tables 9 and 10 for fear and GAD results, respectively); *EA* educational attainment, *SCZ* schizophrenia, *GAD* generalised anxiety disorder, *PTSD* post-traumatic stress disorder, *HDL* high-density lipoprotein cholesterol, *CAD* coronary artery disease, *ADHD* attention deficit hyperactivity disorder.

Three genes were significantly associated with GAD in GAD_anx-dep_ *full* meta-analysis. These were *TMEM106B* on chromosome 7, *YLPM1* on chromosome 14, and *SORCS3* on chromosome 10 (Table 3). The YLPM1 gene was on the same locus as the genome-wide significant SNP-level association with fear (14q24.3). No genes were genome-wide significant in the three other GAD meta-analyses. No gene-sets were significantly associated with GAD after Bonferroni adjustment for 17008 gene-sets tested (*P* ≤ 2.94 × 10^−6^).

### SNP-based heritability

We report results here for the most powerful GWAS (fear_anx-dep_ and GAD_anx-dep_ in the primary *full* meta-analyses), as measured by GWAS mean X^2^. We identified a liability-scale LDSC h^2^_SNP_ for fear_anx-dep_ of 12.9% (SE = 0.9%, mean X^2^ = 1.17) and 12.2% for GAD_anx-dep_ (SE = 0.7%, mean X^2^ = 1.25). We assumed population prevalences of 8.8% for fear and 12.9% for GAD. Supplementary Table 5 reports all h^2^_SNP_ liability-scale estimates, including for alternative population prevalences (±5%), phenotypes with specifically screened controls, and in the *restricted* meta-analysis dataset. Heritability analyses comparing brief versus detailed phenotyping in GLAD+ were underpowered, with not all anxiety phenotypes showing significant h^2^_SNP_ (Supplementary Materials; Supplementary Table 11).

### Global genetic correlation between fear-based disorders and GAD

**Fig. 2:**
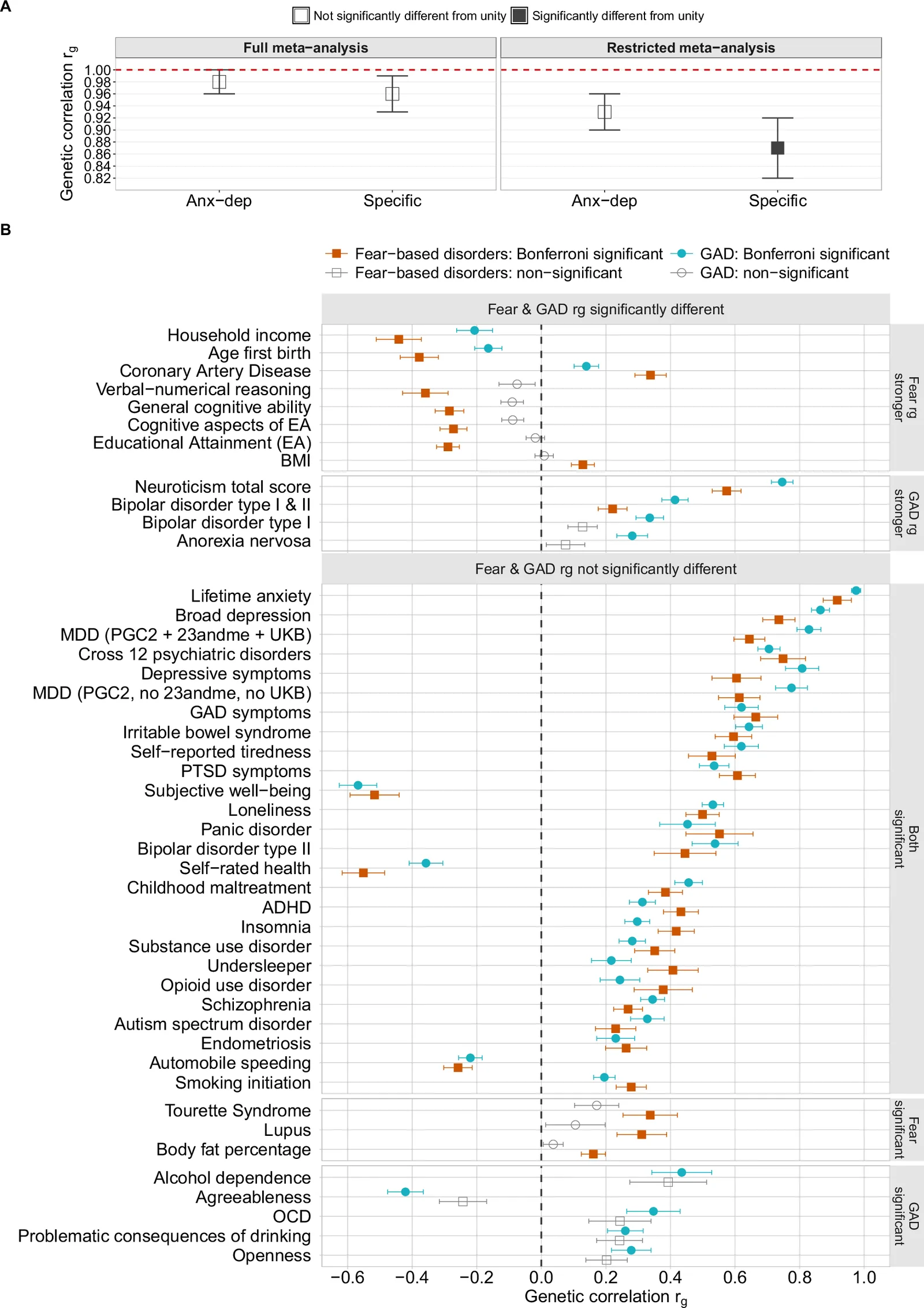
Genetic correlations (r_g_) between fear-based disorders, GAD, and external traits estimated in LDSC regression. **A** Genetic correlation between fear-based disorders and GAD from the Full (GLAD+, UKB & QIMR datasets) and Restricted (GLAD+ and UKB datasets) meta-analyses, using either broadly screened (Anx-dep) or disorder-specific (Specific) controls. The red dashed line indicates unity (r_g_ = 1). Filled squares represent genetic correlations significantly different from unity after Bonferroni correction (P ≤ 0.0125). Hollow squares are not significantly different from unity. Error bars represent standard errors. **B** Fear-GAD genetic correlations with external traits. Fear-based disorder GWAS and GAD GWAS results from restricted meta-analysis with controls screened specifically for their respective anxiety disorders. r_g_ with 106 phenotypes were tested (Bonferroni-corrected significance threshold P ≤ 4.71 × 10^−4^ ; filled shapes: r_g_ significantly different from 0; hollow and grey shapes: r_g_ not significantly different from 0). Differences between the r_g_ of fear-based disorders and that of GAD with each external phenotype were tested using a block jackknife (Bonferroni correction for significance [P ≤ 4.71 × 10^−4^]). Only traits with r_g_ significantly different from 0 with at least one of the anxiety disorder phenotypes are shown and were tested using block jackknife. Full results are shown in Supplementary Table 12. Upper B panel: phenotypes with statistically significant different r_g_ between fear-based disorders and GAD. Lower B panel: phenotypes with no statistically significant different r_g_ between fear-based disorders and GAD, though in the lower two segments, there was a significant association with one trait and not the other. Error bars represent standard errors. ADHD attention deficit hyperactivity disorder, BMI body mass index, GAD generalised anxiety disorder, MDD major depressive disorder, OCD obsessive-compulsive disorder, PGC Psychiatric Genomics Consortium, PTSD post-traumatic stress disorder, UKB UK Biobank.

The global r_g_ between fear and GAD was high (r_g_ = 0.87–0.98) and significantly different from 0 for both fear_specific_–GAD_specific_ and fear_anx-dep_–GAD_anx-dep_ in both primary *full* and secondary *restricted* meta-analysis datasets. Given this high genetic overlap, we also tested whether these correlations significantly differed from unity. We found that only the r_g_ between fear_specific_–GAD_specific_ in *restricted* meta-analyses was significantly less than 1 (i.e., controls were screened specifically and when the depression-enriched QIMR dataset was excluded from the meta-analysis). The other three fear–GAD r_g_ were not significantly different from 1 (Fig. 2A; Bonferroni-corrected significance threshold *P* ≤ 0.0125).

### Global genetic correlations with external phenotypes

We calculated global genetic correlations (r_g_) between fear and GAD with over a hundred external phenotypes. Only fear_specific_ and GAD_specific_ in the *restricted* meta-analyses were tested for r_g_ with external phenotypes (as it was only in this analysis that the fear–GAD r_g_ was significantly different from 1). Of the 106 traits tested, 46 showed significant r_g_ with at least one anxiety phenotype. For most external traits, the r_g_ with fear_specific_ was not significantly different from the r_g_ with GAD_specific_ (Bonferroni-corrected significance threshold *P* ≤ 4.71 × 10^−4^; Fig. 2B; **lower panel**). However, 12 traits showed modest (median |Δr_g_| = 0.20; range = 0.12–0.28), statistically significant differences in their fear-GAD r_g_ (median block-jackknife |Z| = 3.9, range = 3.5–7.7, P ≤ 4.71 × 10⁻⁴). Four cognitive-related traits showed significantly stronger negative r_g_ with fear_specific_ than with GAD_specific_. These included educational attainment (fear-r_g_ = −0.29; GAD-r_g_ = −0.02; |Δr_g_| from unrounded estimates =0.27; jackknife p = 1.55×10^−14^) and general cognitive ability (fear-r_g_ = −0.28; GAD-r_g_ = −0.09; |Δr_g_| = 0.19; p = 4.79×10^−6^). Coronary artery disease showed a higher positive r_g_ with fear_specific_ than with GAD_specific_ (fear-r_g_ = 0.34; GAD-r_g_ = 0.14; |Δrg| = 0.20; p = 3.41 × 10^−6^). Conversely, four traits showed stronger positive r_g_ with GAD_specific_ than with fear_specific_ (Fig. 2B; upper panel). These included bipolar disorder type I (fear-r_g _= 0.13; GAD-r_g_ = 0.34; |Δr_g_| = 0.21; p = 3.39 × 10^−5^), anorexia nervosa (fear-r_g _= 0.07; GAD-r_g _= 0.28; |Δr_g_| = 0.21; p = 4.60 × 10^−4^), and neuroticism total score (fear-r_g _= 0.57; GAD-r_g _= 0.75; |Δr_g_| = 0.17; p = 4.07 × 10^−4^). Full genetic correlation results with external phenotypes are shown in Supplementary Table 12.

### Local genetic correlations

We conducted local r_g_ analyses between fear_specific_–GAD_specific_ (*restricted* meta-analyses) and the 12 external traits showing significantly different fear_specific_–GAD_specific_ global r_g_. We identified 20 significant local r_g_ regions (FDR *q* ≤ 0.1), predominantly with GAD_specific_ (18 regions; Supplementary Tables 13–15). Local-global r_g_ concordance was good for FDR-significant regions (r = 0.8) and moderate for nominally significant regions (r = 0.65). Four regions showed fear-GAD overlap on chromosomes 2 and 7 (r_g_ = 0.96–1.0). Notable genes within these regions include *ZEB2*, *MAGI2*, and *AMPH* (Supplementary Table 14). For external traits, GAD_specific_ had 14 local r_g_ across seven traits, including eight with neuroticism total score (0.69–1.0). Fear_specific_ had two neuroticism local r_g_ (0.87–0.91) at regions also significant for GAD_specific_-neuroticism. One shared fear-GAD-neuroticism region on chromosome 11 contained 19 protein-coding genes (e.g., NCAM1-TTC12-ANKK1-DRD2-HTR3B-HTR3A), and another on chromosome 6 contained 10 protein-coding genes (e.g., *POU3F2*-*SIM1*). Despite non-significant global r_g_, GAD_specific_ had local r_g_ with cognitive aspects of educational attainment (−1.0), BMI (−0.92), and general cognitive ability. For general cognitive ability, two regions showed opposing local r_g_ directions (chromosome 5 = 1.0; chromosome 7 = −1.0). GAD_specific_ showed complete local r_g_ overlap at two regions with bipolar disorder type I-II and anorexia nervosa (both 1.0), consistent with significant global positive r_g_.

### Neuroticism subcomponents

**Fig. 3:**
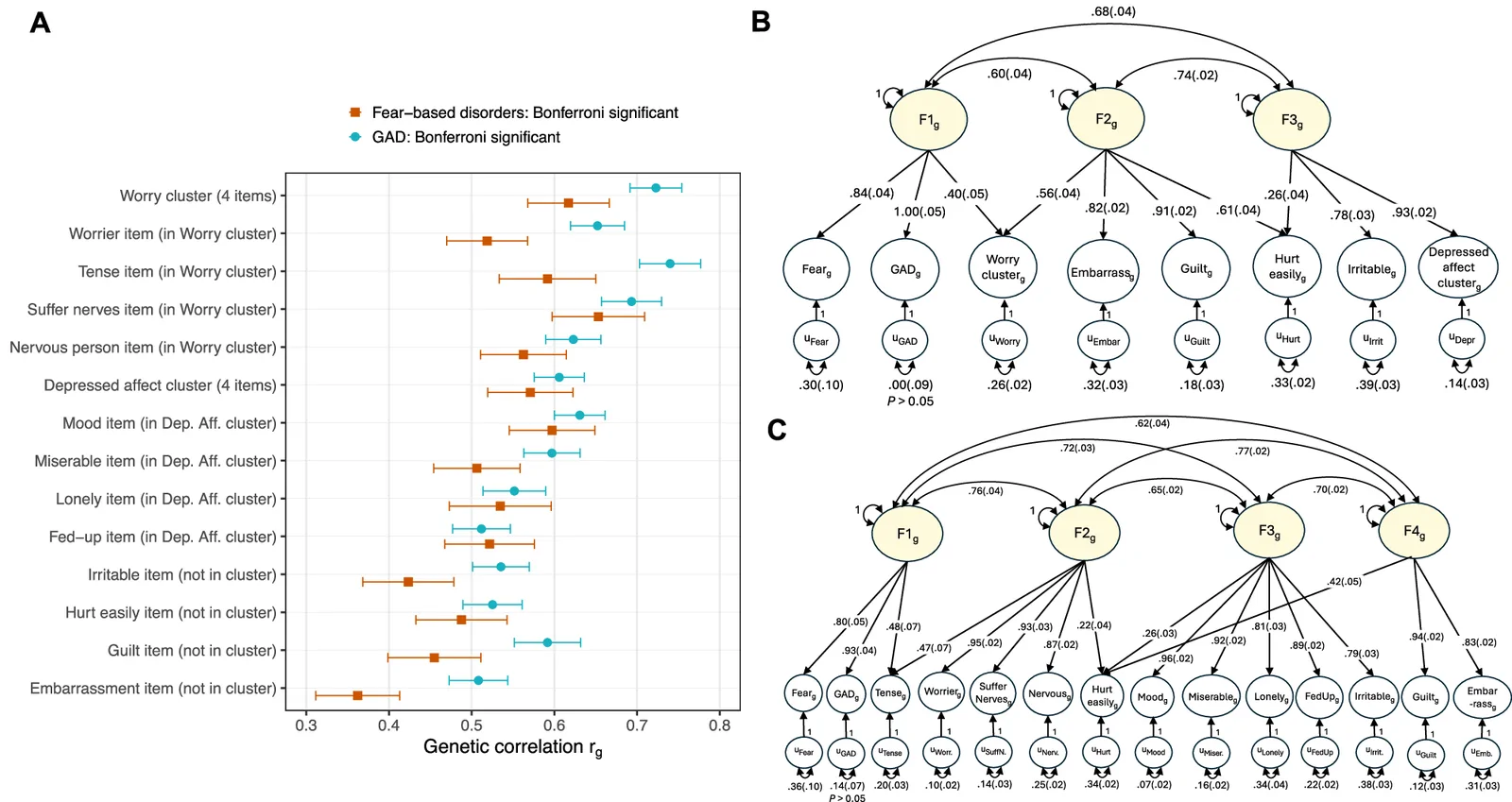
Genetic correlations (r_g_) and genomic factor structure of fear-based disorders, GAD and neuroticism subcomponents. **A** Genetic correlations estimated using LDSC between fear, GAD and neuroticism subcomponents, grouped by previously identified genetic clusters (worry cluster, depressed affect cluster, and unclustered items). Error bars represent standard errors. Filled shapes indicate r_g_ significantly different from 0 (P ≤ 0.0036). **B-C** Genomic SEM standardised estimates for two models: **B** three-factor correlated model of fear, GAD, two neuroticism clusters and four unclustered items, and **C** four-factor correlated model of fear, GAD, and 12 individual neuroticism items. Circles represent the genetic components of phenotypes and the latent genetic factors they load onto. The subscript ‘g’ denotes that variables are defined by their genetic variance as estimated from GWAS used in these models. Single-headed arrows from factor to individual phenotypes represent factor loadings. Double-headed arrows connecting factors represent genetic covariances. Residual variances (u) indicate unique genetic variance not explained by common factors. Standard errors shown in parentheses. P > 0.05 indicates non-significant parameters. GAD = generalised anxiety disorder; LDSC = linkage-disequilibrium score regression; SEM = structural equation modelling.

Although GAD showed a pattern of higher r_g_ than fear across neuroticism subcomponents (Fig. 3A), fear-GAD r_g_ differences were not statistically significant (P ≤ 0.0036; Supplementary Table 16). Using Genomic SEM, we identified a three-factor correlated model encompassing r_g_ between fear, GAD, neuroticism clusters and un-clustered items (excellent fit: AIC = 292, CFI = 0.98, SRMR = 0.031; Supplementary Tables 17–21). The three factors were: (i) anxiety-worry cluster factor with high loadings from GAD and fear, and moderate cross-loadings from worry cluster, (ii) worry-guilt-hurt factor, and (iii) depressed affect-irritability factor (Fig. 3B). Factor analysis of the 12 neuroticism items identified a four-factor correlated model (excellent fit: AIC = 2033, CFI = 0.98, SRMR = 0.048; Supplementary Tables 17 and 22–26). This was consistent with the three-factor cluster model, with fear-GAD loading onto a factor with ‘tense’ worry-related item (Fig. 3C). The remaining three factors aligned with the previously reported neuroticism item clustering: (1) worry, (2) depressed affect, and (3) guilt-embarrassment [58]. In both models (Fig. 3B, C), GAD showed zero residual genetic variance (*P* > 0.05), i.e. the genetic component of GAD was fully explained by the anxiety-worry or anxiety-‘tense’ factor. In contrast, fear retained 30–36% unique genetic variance.

## Discussion

This study reports the first genome-wide significant locus and gene-level associations with any lifetime fear-based disorder diagnosis (fear), and additional loci for GAD. Combining detailed and brief diagnostic measures improved statistical power compared to previous fear GWASs [21, 22]. Our study revealed high genetic correlations between fear, GAD, and depression, consistent with twin studies [10, 12, 13]. Some genetic specificity for fear and GAD emerged after excluding the depression-enriched dataset and screening controls specifically for the anxiety disorder being analysed. This specificity extended to external trait correlations, where fear and GAD showed significantly different genetic relationships with several cognitive, psychiatric, and health-related traits.

Our SNP- and gene-level associations with fear were previously reported in GWASs of educational attainment [61], insomnia [62], major depressive disorder [32] and broadly-defined depression [63], including the *SNX29* gene. The sorting nexin family of proteins is implicated in neuronal function, synaptic plasticity, learning, and memory [64]. These processes are relevant to maladaptive fear learning and conditioning characteristic of fear-based disorders [65]. Two other fear associations (14q24.3 locus and *PAX6* gene) were reported in GWAS of any anxiety disorder [20] and worry-neuroticism [57]. PAX6 regulates brain development, including amygdala formation, with PAX6-deficient rodents showing impaired fear conditioning [66]. Only one fear association (14q24.3) overlapped with GAD (*YLPM1* gene). As GWASs of specific anxiety disorders improve in power, we expect this overlap to increase, and to better discern potential disorder-specific loci. Consistent with shared distress liability, all GAD associations were identified in previous GWASs of distress-related phenotypes [19, 20, 32, 57, 63, 67–69]. This includes the *SORCS3* gene, which is broadly associated with psychiatric phenotypes alongside anxiety [20, 70, 71]. *SORCS3* is highly expressed in the CA1 hippocampus region and implicated in rodent aversive memory extinction [72], a process core to the development and persistence of anxiety disorders [8].

High genetic overlap between fear, GAD, and depression is compatible with twin studies [10, 12, 13] and our preliminary GWASs [22]. We add detail to the genetic structure of fear and GAD by examining their relationship with a wide range of other complex traits. Disorder-specific control screening and excluding the depression-ascertained QIMR dataset improved our ability to identify some genetic specificity between highly genetically correlated disorders. In QIMR, cases had comorbid depression and only broad anxiety control screening was possible. The inclusion of depression-enriched cases and controls screened broadly for other internalising disorders likely elevated shared internalising genetic liability, masking subtle genetic distinctions between fear and GAD. The fear_anx-dep_ 14q24.3 locus reached significance only with broadly-screened controls. This may reflect increased power, or general anxiety-depression liability rather than fear-specific risk. Our findings highlight the importance of careful case-control ascertainment in identifying subtype-specific genetics.

Consistent with a shared internalising genetic liability [10, 12, 13, 73], we found fear and GAD had equivalently high genetic correlations with depression. This common internalising liability factor is often attributed to shared negative affectivity, typically captured by measures of neuroticism [5, 74]. Our neuroticism analyses support twin-based evidence that genetic influences on GAD are more central to higher-order negative affectivity liability than fear [10, 75]. Specifically, we found GAD had a stronger genetic overlap with neuroticism total score than fear. Factor analysis further revealed no remaining genetic influences on GAD beyond those shared with fear alongside either ‘tense’ or the worry cluster. ‘Tense’ as the primary shared fear-GAD neuroticism item suggests physiological tension as a key pathway linking anxiety disorders to trait-like worry. Stronger influences of this pathway on GAD align with muscle tension being a core, discriminative feature of GAD linked to excessive worry [76, 77]. Fear retained substantial genetic influences independent of tense/worry factors, corroborating twin study evidence for partly distinct components from GAD and neuroticism [74]. General negative affectivity also influences fear-based disorders but is less central to them as they are characterised by more specific elements of acute fearful and physiological hyperarousal [4]. Our findings partially support a distress-fear genetic distinction but provide stronger evidence for an overarching internalising genetic liability driving comorbidity across anxiety disorders and depression.

At the regional level, we identified fear-GAD correlated regions with genes involved in inhibitory neurotransmission and synaptic function. *ZEB2* and *AMPH* regulate GABAergic circuitry, with disruption of each producing anxiety-like behaviour in rodent models [78–81]. *MAGI2* maintains glutamatergic transmission through synaptic AMPA receptors, which are involved in fear memory potentiation [82] (Supplementary Discussion). These pathways may underlie the strong global fear-GAD genetic correlation.

Regions shared across fear, GAD and neuroticism contain genes involved in stress response circuitry and monoaminergic signalling. Specifically, on chromosome 6, *POU3F2* and *SIM1* are required for the development of corticotropin-releasing hormone neurons that regulate active versus passive coping during threat [83–85]. Maladaptive threat responses are proposed to underlie anxiety symptoms, such as excessive freezing when active coping would be appropriate [86]. On chromosome 11, *NCAM1* is linked to fear memory [87], dopamine receptor *DRD2* to aversive learning [88], and serotonin receptor subunits (*HTR3A*, *HTR3B)* to stress reactivity [89, 90] (Supplementary Discussion). Dopaminergic and serotonergic signalling are implicated in both internalising and broader neuropsychiatric phenotypes [91–93]. Accordingly, this region was previously identified as a psychiatric pleiotropy hotspot [94], likely capturing broad negative affectivity underlying general psychopathology rather than anxiety-specific mechanisms. Consistent with shared negative affectivity pathways, all fear-neuroticism regions overlapped with GAD-neuroticism. The greater number of GAD-neuroticism regions compared to fear aligns with GAD’s stronger global correlation with neuroticism and greater GWAS power. More powerful GWAS would identify additional fear-GAD regions, enabling fine-mapping to prioritise causal variants and colocalisation to examine whether variants are shared or distinct between phenotypes [95].

Fear’s distinct genetic relationships with cognitive-related traits and heart disease may capture cognitive and autonomic arousal mechanisms less central to GAD. The stronger negative genetic correlations between fear and cognitive-related traits may reflect associative learning, which distinguishes fear-based disorders from GAD [65]. However, GAD’s lack of global correlations with cognitive-related traits may be due to heterogeneous rather than absent shared genetics, given our significant regional genetic correlations in opposite directions. The unique positive genetic correlation of heart disease with fear aligns with a phenotypic study showing that fear-based disorders were stronger predictors of heart disease development than distress disorders [96]. This could be due to earlier onset and longer-term exposure to heart disease risk mechanisms in fear-based disorders [96–98]. Overlapping genetics with heart disease may underpin shared physiological pathways related to inflammation and autonomic dysfunction [99]. Low cardiac vagal tone may mediate anxiety-heart disease associations through maladaptive stress responses, and is more consistently linked with panic disorder than GAD [100, 101]. Symptom overlap (e.g., chest pain) [96] may lead to over-self-reporting of heart disease (e.g., angina) [102] in fear-based disorders, and partly explain our findings.

Subtype-specific influences emerged for bipolar disorder (BD), as GAD showed a stronger genetic correlation than fear with type I (BD-I) but not with type II (BD-II). While fear and GAD comorbidity rates are similar between BD-I and BD-II [103], our genetic findings align with mood disorder genomics research. BD-I, which is characterised by severe manic episodes, is more genetically similar to schizophrenia, whereas BD-II involves hypomania and is more similar both phenotypically and genetically to unipolar depression [77, 104, 105]. As such, the mood disorder spectrum reflects a genetic continuum from manic to hypomanic to depressive symptoms [106]. Our novel finding adds detail to this genetic continuum. Genetically, fear-based disorders lie at the opposite end to BD-I, with distress disorders (GAD and depression) sitting between bipolar disorders and fear.

Despite similar comorbidity rates and proposed shared fear-conditioning mechanisms [107–109], fear showed an unexpectedly weaker genetic correlation with anorexia nervosa than GAD. Previous genetic research on individual anxiety disorders has been restricted to analyses with GAD and anorexia nervosa [110], or other eating disorder phenotypes [111, 112]. A twin study suggested that individual fear disorders vary in their genetic relationships with broadly defined eating disorders [112]. The study found a moderate genetic correlation for panic disorder and lower correlations for phobias and social anxiety disorder [112]. GWAS of individual fear-based disorders would enable testing of whether genetic correlations with eating disorder phenotypes differ across fear subtypes, and whether this corroborates the twin-based results.

Our findings should be considered in light of limitations. First, we were underpowered to assess individual fear-based disorders, which may have varying disorder-specific genetic influences as indicated by twin studies [12, 13]. Second, combining panic disorder diagnosis with panic attack measures might have captured broad psychopathology genetics, given that panic attacks are transdiagnostic [77]. Third, phenotyping varied across datasets, with no detailed fear measures in UKB MHQ1 and only broad anxiety control measures in QIMR. Finally, several factors may have elevated genetic correlations between fear, GAD and related disorders like depression. Inclusion of a small proportion of cases from the eating disorder-enriched EDGI UK study may have increased genetic overlap with anorexia nervosa. By combining detailed with brief measures we may have limited genetic specificity [28]. Similarly, we could not account for high lifetime comorbidity across anxiety disorders and depression. In the GLAD+ dataset, only 5% of cases met criteria for a single anxiety disorder without comorbid MDD [3]. Ascertaining data for a well-powered GWAS of a single anxiety disorder without comorbidities will be challenging, given the considerable clinical overlap between anxiety disorders. Such samples would also be less representative of the broader lived experience of people with these disorders. Expanding datasets with detailed diagnostic and dimensional phenotyping of all anxiety disorders, via additional recruitment (e.g., GLAD+, AGDS) or phenotyping existing biobanks, will be key to identifying subtype-specific genetic factors.

In summary, our results provide further evidence for shared genetic liability between fear-based disorders, GAD, and depression, while revealing some genetic specificity. High fear-GAD genetic correlations support the ‘any anxiety’ GWAS approach to maximise power, whilst evidence of some genetic specificity with refined phenotyping warrants further subtype-specific GWAS. Genes associated with fear and GAD point to the neurobiology of threat learning and stress response, which may contribute to their phenotypic similarity. In terms of genetic specificity, genetic factors associated with muscle tension and negative affect appear more central to GAD, while those linked to acute physiological arousal are implicated in fear. Such divergent factors may underlie the distinct clinical presentations between fear and GAD. Our findings add more fine-grained detail to the proposed hierarchical structure of internalising disorders and partially support fear-based disorders and GAD as separate distress-fear subtypes. Quantitatively based GWAS [71] and dimensional modelling of distress-fear symptoms would reveal shared and distinct genomics at narrower levels of this hierarchical structure [5].

## Supplementary information

Supplementary Material

Supplementary Tables

## Data Availability

Fear and GAD GWAS summary statistics have been deposited on Zenodo at https://doi.org/10.5281/zenodo.17228387. Access to the individual-level data used in this study is subject to approval by the respective data access committees and can be requested as follows: GLAD, COPING and EDGI UK through the NIHR BioResource at https://www.bioresource.nihr.ac.uk/using-our-bioresource/apply-for-bioresource-data-access; UK Biobank at https://www.ukbiobank.ac.uk/enable-your-research/apply-for-access/; AGDS data access requests should be directed to N.G.M. (nick.martin@qimrberghofer.edu.au).

## Code availability

The analyses were conducted using the following publicly available software: REGENIE (v3.1.3) (https://github.com/rgcgithub/regenie), SAIGE (v0.44) (https://github.com/weizhouUMICH/SAIGE), METAL (2020-05-05 release) (https://github.com/statgen/METAL), MungeSumstats (v1.10.1) (https://github.com/Al-Murphy/MungeSumstats), FUMA (v1.5.2) (https://fuma.ctglab.nl/), LDSC (v1.0.1) (https://github.com/bulik/ldsc), LAVA (v0.1.5) (https://github.com/josefin-werme/LAVA), GenomicSEM (v0.0.5) (https://github.com/GenomicSEM/GenomicSEM), and biomaRt (v2.64.0) (https://github.com/Huber-group-EMBL/biomaRt).
