# Supplementary material for: Genome-wide genetic overlap between fear-based disorders and generalised anxiety disorder
Source: Mol Psychiatry. 2026 Jun 29;31(11):6224–37. doi: 10.1038/s41380-026-03677-2 (PMC13668672; doi:10.1038/s41380-026-03677-2)
Supplement: Supplementary file 1 [file 41380_2026_3677_MOESM1_ESM.docx]

Supplementary materials for “Genome-wide genetic overlap between fear-based disorders and generalised anxiety disorder”

# Table of Contents

**Supplementary Methods	2**

Study recruitment, genotyping and quality control	2

GLAD+	2

UK Biobank	2

QIMR Berghofer	3

Processing of GWAS summary statistics for harmonisation	4

Comparing fear-GAD genetic correlations using block-jackknife and LDSC	4

Local genetic correlations	5

Neuroticism follow-up analyses	5

SNP-based heritability estimation of detailed and brief phenotyping of fear and GAD	5

**Supplementary Results	7**

GWAS meta-analyses in the full datasets (GLAD+, QIMR and UKB)	7

Supplementary Figure 1: Q-Q plots	7

Supplementary Figure 2: Manhattan plots	8

GWAS meta-analyses in the restricted datasets (GLAD+ and UKB, excluding QIMR)	9

Supplementary Figure 3: Q-Q plots	9

Supplementary Figure 4: Manhattan plots	10

Region plots of genome-wide significant loci	12

Fear-based results	12

Supplementary Figure 5: Locus on 14q24.3	12

GAD results	12

Supplementary Figure 6: Locus on 9p23	12

Supplementary Figure 7: Locus on 6p22.1	13

Supplementary Figure 8: Locus on 6q16.3	13

Supplementary Figure 9: Locus on 2p16.1	14

Neuroticism subcomponents analysis: genetic correlation matrices	15

Supplementary Figure 10: Genetic correlation matrix for fear, GAD, neuroticism clusters and unclustered items	15

Supplementary Figure 11: Genetic correlation matrix for fear, GAD, and 12 neuroticism items	16

**Supplementary Discussion	17**

Biological interpretation of fear-GAD local genetic correlation regions	17

Biological interpretation of fear-GAD-neuroticism local genetic correlation regions	17

Chromosome 11	17

Chromosome 6	18

**References	19**

# Supplementary Methods

## Study recruitment, genotyping and quality control

### GLAD+

The GLAD Study is an ongoing online research platform launched in September 2018 to recruit participants with lifetime anxiety and/or depressive disorder. GLAD recruitment is open to individuals aged 16 years or older residing in the UK. Initial recruitment involved a widespread media campaign, followed by ongoing social media advertising and participating NHS trusts and GP practices [1]. The EDGI UK Study was established in 2020 with ongoing recruitment of UK-based participants aged 16+ with eating disorders through online platforms and clinical services [2]. The COPING study is a longitudinal survey collecting data within GLAD, EDGI UK and other NIHR BioResource studies [3], including a proportion of participants recruited through an inflammatory bowel disease study [4].

In the GLAD, COPING and EDGI UK studies, genotyping was performed using the Affymetrix UK Biobank Axiom Array v1 and v2. Participants were defined as having European-associated ancestry based on genomic principal component analysis (PCA) conducted in Plink. Genotyping quality control was performed on the PCA-defined European-associated ancestry subsample. Pre-imputed variants that were rare (MAF < 1%), not present in > 95% of participants, and showed deviation from the Hardy‐Weinberg equilibrium (HWE; p < 1x 10-10) were removed. Individuals were excluded if they had > 95% of variants missing, a genetic duplicate across the two studies, a mismatch between self-reported and chromosomal sex and were outliers of identity by descent, indicating sample contamination (> 3 standard deviations from the mean). We excluded 138 SNPs that showed saliva-specific associations when comparing saliva-derived (GLAD+) versus blood-derived genotyping (UK Biobank) datasets. To prevent duplication of individuals in the GWAS meta-analysis, we excluded 1491 GLAD+ participants who were also participants of the UK Biobank. Principal components used as GWAS covariates were derived by projecting the GLAD+ genotype data onto the UK Biobank principal component space to account for population stratification. Genotyping batch and array were included as additional covariates to control for technical variation.

### UK Biobank

The UK Biobank is a population-based cohort of over 500,000 participants aged 40-69 recruited between 2006 and 2010 [4]. In 2017, a subset of participants (N = 157,366) completed an online follow-up Mental Health Questionnaire assessing mental health and well-being through self-report measures [5].

Pre-processing and collection of the UK Biobank genotype data was conducted centrally as described elsewhere [5]. Participants were genotyped using the Affymetric UK BiLEVE Axiom and Affymetric UK Biobank Axiom array. Participants were defined as having European-associated ancestry based on genomic PCA four-means clustering. In the PCA-defined European-associated ancestry dataset, pre-imputed variants with MAF < 1%, missing in > 98% of individuals, and significant results from the HWE deviation test (p < 1 × 10^−8^ ). Participants were removed if they had > 98% of variants missing and a mismatch between genetic and phenotypic sex.

### QIMR Berghofer

The Queensland Institute of Medical Research (QIMR) Berghofer dataset comprised the Australian Genetics of Depression Study (AGDS) [6] and the QIMR Berghofer QSkin Sun & Health Study [7]. AGDS was launched in September 2016 to collect genetic and phenotypic data from individuals with lifetime depression, with recruitment ongoing through pharmaceutical prescription history provided by the Australian government and media campaigns. Participation is open to individuals aged 18 years or older across Australia [6]. The QSkin Sun & Health Study is a population-based study established in 2010 of over 40,000 Queensland residents aged 40-69 years, initially focused on skin cancer research, with participants later completing brief self-report measures for mental health conditions [7].

Participants in the AGDS and QIMR studies were genotyped using the Illumina Global Screening Array v2. Genotyping quality control was performed in a PCA-defined European-associated ancestry subsample, as described elsewhere [8]. Briefly, prior to imputation, variants were removed with MAF < 1%, high missingness > 95% and deviation from the HWE (p < 1 × 10^‐6^).

Genome-wide association analyses

Logistic mixed model analyses were performed using the software REGENIE for analyses in the GLAD+ dataset and SAIGE in the AGDS dataset. Logistic regression score tests were applied to account for unbalanced case-control ratios, with approximate Firth logistic regression test used in REGENIE and the saddle point approximation test used in SAIGE [9, 10].

## Processing of GWAS summary statistics for harmonisation

We used the Bioconductor R package MungeSumstats [11] to harmonise GWAS summary statistics to GRCh38 reference genome before meta-analysis, using dbSNP build 155 (package 'SNPlocs.Hsapiens.dbSNP155.GRCh38') and NCBI reference genome (package 'BSgenome.Hsapiens.NCBI.GRCh38'). For the UK Biobank dataset, this included genome build coordinate conversion from GRCh37 to GRCh38 using Ensembl genomic mapping files. Quality control included harmonising SNP IDs against the reference genome, imputing missing RSIDs from dbSNP 155 using chromosomal positions, and removing duplicated variants and those with zero standard errors. To maintain consistent effect allele designation, alleles were checked against the reference genome, and effect allele, effect estimates and frequencies were flipped where needed to match the reference. Variants missing from the reference were excluded. After meta-analysis with METAL, which can reorder effect and non-effect alleles to alphabetical ordering, we used MungeSumstats again to preserve effect allele alignment with the reference genome.

To prepare data for LDSC regression and Genomic SEM analyses, GWAS summary statistics were processed using the LDSC munge function with default parameters [12]. This included filtering to SNPs within the HapMap 3 reference panel with INFO > 0.9 and MAF > 0.01.

## Comparing fear-GAD genetic correlations using block-jackknife and LDSC

The block-jackknife method provides robust standard errors that can be used to formally test whether genetic correlations differ significantly between two pairs of phenotypes [13, 14]. For our analysis comparing fear-external trait correlations to GAD-external trait correlations, we used LDSC software which outputs jackknife delete values for genetic covariance and heritability [15]. These delete values are calculated by excluding blocks of SNPs (n blocks = 200). From these outputs, we calculated genetic correlation delete values for each block exclusion [13, 14]. The difference between the global genetic correlation estimates (fear-external trait minus GAD-external trait) provides the overall difference, while the differences between the delete value vectors provide the block-specific differences. We then computed jackknife pseudovalues for each block i as: n × (global difference) - (n-1) × (block i difference). The mean of these pseudovalues provides the jackknife estimate of the correlation difference, and the variance of the pseudovalues is used to calculate the standard error. Finally, we derived a z-statistic by dividing the mean pseudovalue by the standard error of the mean (√((1/n) × variance)), which tests the null hypothesis that the two genetic correlations are equal [13, 14]. P-values were calculated from this z-statistic, with significance determined after stringent Bonferroni correction (p < 4.71 × 10⁻⁴).

## Local genetic correlations

We conducted local genetic correlation analyses using LAVA [16] to better characterise global r_g_s by identifying region-specific genetic relationships between fear_specific_ and GAD_specific_, and their relationship with external traits. We limited analyses with external traits to those showing significantly different fear_specific_–GAD_specific_ global r_g_s, as these traits are most informative for identifying genomic regions that may distinguish Fear from GAD. This approach also reduced the multiple testing burden, given the limited statistical power in our anxiety GWASs. To further address limited statistical power for LAVA analyses, we made several additional adjustments. First, the genomic window size was increased from the original LAVA default of 2,500 variants to 5,000 variants, resulting in 1,226 genomic blocks [16]. Second, we used a more liberal univariate heritability threshold (*P* ≤ 0.05) than in the original methods paper [16], an approach supported by the developer of the method, as the Type 1 error rate of the subsequent bivariate test is independent of this initial filtering stringency [16, 17]. Third, to define significant bivariate tests, we applied FDR correction at q < 0.1 rather than q < 0.05. This more liberal threshold has been applied in previous local genetic correlation analyses [18]. We used the UK Biobank-derived LAVA LD reference panel to reduce inflated Type 1 error rates and biased local heritability estimates observed in smaller reference samples [19]. We annotated protein-coding genes within each significant region using genomic coordinates from Ensembl and the biomaRt R package [20, 21].

## Neuroticism follow-up analyses

We analysed two previously identified neuroticism genetic clusters (worry and depressed affect) [22, 23] and 12 individual items from the UKB Eysenck Personality Questionnaire [23]. For Genomic SEM, exploratory factor analysis (EFA) results using promax (correlated) rotations were used to construct the genetic factor models. Phenotypes with standardised factor loadings >0.25 were retained for the corresponding latent factors, with cross-loadings permitted where indicated. Second, confirmatory factor analysis (CFA) models based on the EFA results were fitted using diagonally weighted least squares (DWLS) estimation. Models were compared using the Akaike information criterion (AIC), comparative fit index (CFI), and standardised root mean square residual (SRMR). Model fit was considered excellent when CFI ≥ 0.95 and SRMR ≤ 0.05, and acceptable when CFI ≥ 0.90 and SRMR ≤ 0.10 [24].

## SNP-based heritability estimation of detailed and brief phenotyping of fear and GAD

We estimated the h^2^_SNP_ of GAD and the fear-based disorder, where we screened cases and controls using only i) detailed symptom-based measures ii) brief self-report measures. Analyses were restricted to the GLAD+ dataset, where both diagnostic measures were available to screen cases and controls for all five anxiety disorders. Controls were specifically screened for the disorder being analysed. GCTA-GREML was used to estimate h^2^_SNP_ on the liability scale using individual-level data [25]). Sample sizes were underpowered to estimate h^2^_SNP_ in LDSC regression (Supplementary Table 11).

# Supplementary Results

## GWAS meta-analyses in the full datasets (GLAD+, QIMR and UKB)

### Supplementary Figure 1: Q-Q plots

Q-Q plots of GWAS of fear and GAD in the full meta-analysis datasets.

### Supplementary Figure 2: Manhattan plots

Manhattan plots for GWAS of GAD and fear with specifically screened controls in the full meta-analysis datasets (GLAD+, QIMR and UK). Line red; common genetic variant genome-wide significance threshold (P ≤ 5 × 10^−8^), blue; suggestive significance threshold (P ≤ 1 × 10^−5^).

## GWAS meta-analyses in the restricted datasets (GLAD+ and UKB, excluding QIMR)

### Supplementary Figure 3: Q-Q plots

Q-Q plots for GWAS of GAD and fear in the restricted meta-analysis datasets.

### Supplementary Figure 4: Manhattan plots

Manhattan plots for GWAS of GAD and fear in the restricted meta-analysis datasets. Line red; common genetic variant genome-wide significance threshold (P ≤ 5 × 10^−8^), blue; suggestive significance threshold (P ≤ 1 × 10^−5^).

## Region plots of genome-wide significant loci

### Fear-based results

#### Supplementary Figure 5: Locus on 14q24.3

Regional plot for the *14q24.3* locus shown for Fear GWAS (anx-dep controls in the full meta-analysis).

### GAD results

#### Supplementary Figure 6: Locus on 9p23

Regional plot for the 9p23 locus shown for the most well-powered GAD GWAS (anx-dep controls in the full meta-analysis).

#### Supplementary Figure 7: Locus on 6p22.1

Regional plot for the 6p22.1 locus shown for the most well-powered GAD GWAS (anx-dep controls in the full meta-analysis).

#### Supplementary Figure 8: Locus on 6q16.3

Regional plot for the 6q16.3 locus shown for GAD GWAS (anx-dep controls in the full meta-analysis).

#### Supplementary Figure 9: Locus on 2p16.1

Regional plot for the 2p16.1 locus shown for GAD GWAS (specific controls in the full meta-analysis).

## Neuroticism subcomponents analysis: genetic correlation matrices

#### Supplementary Figure 10: Genetic correlation matrix for fear, GAD, neuroticism clusters and unclustered items

*Genetic correlations estimated using LDSC between fear-based disorders, generalised anxiety disorder, two neuroticism genetic clusters (worry cluster, depressed affect cluster), and four unclustered neuroticism items (guilt, hurt easily, irritable, embarrassed). This genetic correlation matrix serves as input for genomic structural equation modelling analyses (Supplementary Tables 18-21).*

#### Supplementary Figure 11: Genetic correlation matrix for fear, GAD, and 12 neuroticism items

Genetic correlations estimated using LDSC between fear-based disorders, generalised anxiety disorder, and 12 individual neuroticism items. *This genetic correlation matrix serves as input for genomic structural equation modelling analyses (Supplementary Tables 22-26).*

# Supplementary Discussion

## Biological interpretation of fear-GAD local genetic correlation regions

One chromosome 2 fear-GAD region contains *ZEB2*, which regulates the development and positioning of GABAergic interneurons in the cortex and hippocampus [26]. Heterozygous *ZEB2* mutant mice show increased anxiety, reduced motor activity, and fewer inhibitory interneurons in the cortex [27]. One chromosome 7 fear-GAD region contains *MAGI2*, which encodes a scaffolding protein that maintains AMPA receptor density at hippocampal synapses [28]. Stress-induced AMPA receptor remodelling can enhance fear memory [29], suggesting *MAGI2* variation could influence fear and anxiety phenotypes through altered synaptic plasticity. The other chromosome 7 fear-GAD region contains *AMPH*, which encodes amphiphysin, a presynaptic scaffolding protein essential for sustained GABA release [30]. Disruption of amphiphysin function impairs GABAergic inhibition and produces anxiety-like behaviour in rodents [30, 31].

These regions also contained genes with less direct evidence linking them to fear or anxiety. For example, the chromosome 7 *MAGI2* region contains *YWHAG*, which encodes the 14-3-3γ signalling protein. Mice with reduced 14-3-3γ levels show stress-sensitive behaviour and neurodevelopmental delay [32], and human *YWHAG* mutations cause a neurodevelopmental syndrome frequently accompanied by behavioural and psychiatric comorbidities [33]. A second chromosome 2 region contains *KCNH7*, which encodes a voltage-gated potassium channel (Kv11.3) that stabilises neuronal excitability in cerebellar Purkinje cells [34]. *KCNH7* knockout mice display depressive-like behaviour [34], and reduced *KCNH7* expression in anterior cingulate interneurons is associated with social interaction deficits [35]. However, the primary phenotypes reported for these genes relate to depression, social cognition, and neurodevelopmental syndromes rather than fear or anxiety specifically.

## Biological interpretation of fear-GAD-neuroticism local genetic correlation regions

### Chromosome 11

The chromosome 11 region showing shared local genetic correlation between fear, GAD and neuroticism contained 19 protein-coding genes, including several with roles in threat-related brain function. This region overlaps with the *NCAM1-TTC12-ANKK1-DRD2* (NTAD) cluster, previously identified as a highly pleiotropic region shared across multiple psychiatric disorders [36]. The genes within this cluster have been suggested to show coordinated regulation and function [37, 38].

*NCAM1* encodes neural cell adhesion molecule, which is highly expressed in the amygdala, where it supports synaptic remodelling for fear memory consolidation [39]. *NCAM1*-deficient mice show impaired fear memory and reduced synchronised activity between the amygdala and hippocampus during fear retrieval [39, 40].

*DRD2* encodes the dopamine D2 receptor, expressed in the bed nucleus of the stria terminalis (BNST) and amygdala, where it regulates the ability to distinguish between threat and safety cues [41]. D2 receptor inhibition in the BNST and amygdala causes fear overgeneralisation in mice, whilst D2 receptor activation improves threat-safety discrimination [41]. *ANKK1* encodes a signalling kinase that regulates dopamine D2 receptor activity [42]. *ANKK1*-deficient zebrafish show disrupted dopaminergic pathways [42]. *TTC12* has been tentatively linked to dopaminergic neurodevelopment via the Wnt signalling pathway [37],

*HTR3A* and *HTR3B* encode subunits of the serotonin 5-HT3 receptor. The 5-HT3 receptor is expressed on GABAergic interneurons in the amygdala and other limbic regions involved in threat processing *[43]*. Mice lacking functional 5-HT3 receptors show reduced anxiety-like behaviour [44, 45] and reduced stress-induced activation of the hypothalamic-pituitary-adrenal axis [46].

### Chromosome 6

The chromosome 6 region with shared fear-GAD-neuroticism local genetic correlation contained 10 protein-coding genes. This includes *POU3F2* (also known as BRN2) and *SIM1,* which both contribute to the formation of the hypothalamic paraventricular nucleus (PVN), the brain region that initiates the hormonal stress response [47]. *SIM1* maintains *POU3F2* expression during PVN development, and loss of function of either gene prevents the formation of neurons that produce corticotropin-releasing hormone (CRH) in the PVN [47]. CRH neurons in the PVN initiate the cascade of stress hormone release and regulate the selection between active (e.g., escape) and passive (e.g., freezing) coping behaviours during threat responses in animal models [48, 49]. Maladaptive shifts between active and passive defensive strategies are proposed to underlie anxiety symptoms [50]. For example, an individual with anxiety might exhibit excessive passive freezing or avoidance when active coping would be more appropriate [50].

# References

1.	Davies MR, Kalsi G, Armour C, Jones IR, McIntosh AM, Smith DJ, et al. The Genetic Links to Anxiety and Depression (GLAD) Study: Online recruitment into the largest recontactable study of depression and anxiety. Behav Res Ther. 2019;123:103503.

2.	Monssen D, Davies HL, Kakar S, Bristow S, Curzons SCB, Davies MR, et al. The United Kingdom Eating Disorders Genetics Initiative. Int J Eat Disord. 2023. 16 August 2023. https://doi.org/10.1002/eat.24037.

3.	Young KS, Purves KL, Hübel C, Davies MR, Thompson KN, Bristow S, et al. Depression, anxiety and PTSD symptoms before and during the COVID-19 pandemic in the UK. Psychol Med. 2022:1–14.

4.	Davies HL, Hübel C, Herle M, Kakar S, Mundy J, Peel AJ, et al. Risk and protective factors for new-onset binge eating, low weight, and self-harm symptoms in> 35,000 individuals in the UK during the COVID-19 pandemic. Int J Eat Disord. 2022. 2022.

5.	Bycroft C, Freeman C, Petkova D, Band G, Elliott LT, Sharp K, et al. The UK Biobank resource with deep phenotyping and genomic data. Nature. 2018;562:203–209.

6.	Byrne EM, Kirk KM, Medland SE, McGrath JJ, Colodro-Conde L, Parker R, et al. Cohort profile: the Australian genetics of depression study. BMJ Open. 2020;10:e032580.

7.	Olsen CM, Green AC, Neale RE, Webb PM, Cicero RA, Jackman LM, et al. Cohort profile: the QSkin Sun and Health Study. Int J Epidemiol. 2012;41:929–929i.

8.	Mitchell BL, Campos AI, Whiteman DC, Olsen CM, Gordon SD, Walker AJ, et al. The Australian Genetics of Depression Study: New Risk Loci and Dissecting Heterogeneity Between Subtypes. Biol Psychiatry. 2021. 2 November 2021. https://doi.org/10.1016/j.biopsych.2021.10.021.

9.	Mbatchou J, Barnard L, Backman J, Marcketta A, Kosmicki JA, Ziyatdinov A, et al. Computationally efficient whole-genome regression for quantitative and binary traits. Nat Genet. 2021;53:1097–1103.

10.	Zhou W, Nielsen JB, Fritsche LG, Dey R, Gabrielsen ME, Wolford BN, et al. Efficiently controlling for case-control imbalance and sample relatedness in large-scale genetic association studies. Nat Genet. 2018;50:1335–1341.

11.	Murphy AE, Schilder BM, Skene NG. MungeSumstats: a Bioconductor package for the standardization and quality control of many GWAS summary statistics. Bioinformatics. 2021;37:4593–4596.

12.	Bulik-Sullivan BK, Loh P-R, Finucane HK, Ripke S, Yang J, Schizophrenia Working Group of the Psychiatric Genomics Consortium, et al. LD Score regression distinguishes confounding from polygenicity in genome-wide association studies. Nat Genet. 2015;47:291–295.

13.	Hübel C, Gaspar HA, Coleman JRI, Finucane H, Purves KL, Hanscombe KB, et al. Genomics of body fat percentage may contribute to sex bias in anorexia nervosa. Am J Med Genet B Neuropsychiatr Genet. 2019;180:428–438.

14.	Coleman JRI, Gaspar HA, Bryois J, Bipolar Disorder Working Group of the Psychiatric Genomics Consortium, Major Depressive Disorder Working Group of the Psychiatric Genomics Consortium, Breen G. The Genetics of the Mood Disorder Spectrum: Genome-wide Association Analyses of More Than 185,000 Cases and 439,000 Controls. Biol Psychiatry. 2019. 1 November 2019. https://doi.org/10.1016/j.biopsych.2019.10.015.

15.	Bulik-Sullivan B, Finucane HK, Anttila V, Gusev A, Day FR, Loh P-R, et al. An atlas of genetic correlations across human diseases and traits. Nat Genet. 2015;47:1236–1241.

16.	Werme J, van der Sluis S, Posthuma D, de Leeuw CA. An integrated framework for local genetic correlation analysis. Nat Genet. 2022;54:274–282.

17.	de Leeuw CA. Possibility to use a less strict P value threshold for univariate analysis. LAVA GitHub Issues. 2022. https://github.com/josefin-werme/LAVA/issues/38. Accessed 1 August 2025.

18.	Zhang C, Zhang Y, Zhang Y, Zhao H. Benchmarking of local genetic correlation estimation methods using summary statistics from genome-wide association studies. Brief Bioinform. 2023;24:bbad407.

19.	de Leeuw C, Werme J, Posthuma D. Re-evaluating local genetic correlation analysis in the HDL framework. Preprints. 2025.

20.	Durinck S, Moreau Y, Kasprzyk A, Davis S, De Moor B, Brazma A, et al. BioMart and Bioconductor: a powerful link between biological databases and microarray data analysis. Bioinformatics. 2005;21:3439–3440.

21.	Durinck S, Spellman PT, Birney E, Huber W. Mapping identifiers for the integration of genomic datasets with the R/Bioconductor package biomaRt. Nat Protoc. 2009;4:1184–1191.

22.	Nagel M, Jansen PR, Stringer S, Watanabe K, de Leeuw CA, Bryois J, et al. Meta-analysis of genome-wide association studies for neuroticism in 449,484 individuals identifies novel genetic loci and pathways. Nat Genet. 2018;50:920–927.

23.	Nagel M, Watanabe K, Stringer S, Posthuma D, van der Sluis S. Item-level analyses reveal genetic heterogeneity in neuroticism. Nat Commun. 2018;9:905.

24.	Grotzinger AD, Rhemtulla M, de Vlaming R, Ritchie SJ, Mallard TT, Hill WD, et al. Genomic structural equation modelling provides insights into the multivariate genetic architecture of complex traits. Nat Hum Behav. 2019;3:513–525.

25.	Yang J, Lee SH, Goddard ME, Visscher PM. GCTA: a tool for genome-wide complex trait analysis. Am J Hum Genet. 2011;88:76–82.

26.	Epifanova E, Babaev A, Newman AG, Tarabykin V. Role of Zeb2/Sip1 in neuronal development. Brain Res. 2019;1705:24–31.

27.	Takagi T, Nishizaki Y, Matsui F, Wakamatsu N, Higashi Y. De novo inbred heterozygous Zeb2/Sip1 mutant mice uniquely generated by germ-line conditional knockout exhibit craniofacial, callosal and behavioral defects associated with Mowat-Wilson syndrome. Hum Mol Genet. 2015;24:6390–6402.

28.	Danielson E, Zhang N, Metallo J, Kaleka K, Shin SM, Gerges N, et al. S-SCAM/MAGI-2 is an essential synaptic scaffolding molecule for the GluA2-containing maintenance pool of AMPA receptors. J Neurosci. 2012;32:6967–6980.

29.	Yang Q, Abdulla A, Farooq M, Ishikawa Y, Liu SJ. Emotional stress increases GluA2 expression and potentiates fear memory via adenylyl cyclase 5. Cell Rep. 2025;44:115180.

30.	Geis C, Weishaupt A, Hallermann S, Grünewald B, Wessig C, Wultsch T, et al. Stiff person syndrome-associated autoantibodies to amphiphysin mediate reduced GABAergic inhibition. Brain. 2010;133:3166–3180.

31.	Geis C, Grünewald B, Weishaupt A, Wultsch T, Toyka KV, Reif A, et al. Human IgG directed against amphiphysin induces anxiety behavior in a rat model after intrathecal passive transfer. J Neural Transm (Vienna). 2012;119:981–985.

32.	Kim DE, Cho C-H, Sim KM, Kwon O, Hwang EM, Kim H-W, et al. 14-3-3γ haploinsufficient mice display hyperactive and stress-sensitive behaviors. Exp Neurobiol. 2019;28:43–53.

33.	Cetica V, Pisano T, Lesca G, Marafi D, Licchetta L, Riccardi F, et al. Clinical and molecular characterization of patients with YWHAG-related epilepsy. Epilepsia. 2024;65:1439–1450.

34.	Schwarz JR, Freitag S, Pechmann Y, Hermans-Borgmeyer I, Wagner W, Hornig S, et al. Purkinje cell hyperexcitability and depressive-like behavior in mice lacking erg3 (ether-à-go-go-related gene) K+ channel subunits. Sci Adv. 2024;10:eadn6836.

35.	Qi C, Sima W, Mao H, Hu E, Ge J, Deng M, et al. Anterior cingulate cortex parvalbumin and somatostatin interneurons shape social behavior in male mice. Nat Commun. 2025;16:4156.

36.	Grotzinger AD, Werme J, Peyrot WJ, Frei O, de Leeuw C, Bicks LK, et al. Mapping the genetic landscape across 14 psychiatric disorders. Nature. 2026;649:406–415.

37.	Mota NR, Araujo-Jnr EV, Paixão-Côrtes VR, Bortolini MC, Bau CHD. Linking dopamine neurotransmission and neurogenesis: the evolutionary history of the NTAD (NCAM1-TTC12-ANKK1-DRD2) gene cluster. Genet Mol Biol. 2012;35:912–918.

38.	Huang W, Payne TJ, Ma JZ, Beuten J, Dupont RT, Inohara N, et al. Significant association of ANKK1 and detection of a functional polymorphism with nicotine dependence in an African-American sample. Neuropsychopharmacology. 2009;34:319–330.

39.	Albrecht A, Bergado-Acosta JR, Pape H-C, Stork O. Role of the neural cell adhesion molecule (NCAM) in amygdalo-hippocampal interactions and salience determination of contextual fear memory. Int J Neuropsychopharmacol. 2010;13:661–674.

40.	Bisaz R, Sandi C. The role of NCAM in auditory fear conditioning and its modulation by stress: a focus on the amygdala. Genes Brain Behav. 2010;9:353–364.

41.	De Bundel D, Zussy C, Espallergues J, Gerfen CR, Girault J-A, Valjent E. Dopamine D2 receptors gate generalization of conditioned threat responses through mTORC1 signaling in the extended amygdala. Mol Psychiatry. 2016;21:1545–1553.

42.	Leggieri A, García-González J, Torres-Perez JV, Havelange W, Hosseinian S, Mech AM, et al. Ankk1 loss of function disrupts dopaminergic pathways in zebrafish. Front Neurosci. 2022;16:794653.

43.	Fakhfouri G, Rahimian R, Dyhrfjeld-Johnsen J, Zirak MR, Beaulieu J-M. 5-HT3 receptor antagonists in neurologic and neuropsychiatric disorders: The iceberg still lies beneath the surface. Pharmacol Rev. 2019;71:383–412.

44.	Martin V, Riffaud A, Marday T, Brouillard C, Franc B, Tassin J-P, et al. Response of Htr3a knockout mice to antidepressant treatment and chronic stress. Br J Pharmacol. 2017;174:2471–2483.

45.	Kelley SP, Bratt AM, Hodge CW. Targeted gene deletion of the 5-HT3A receptor subunit produces an anxiolytic phenotype in mice. Eur J Pharmacol. 2003;461:19–25.

46.	Bhatnagar S, Sun LM, Raber J, Maren S, Julius D, Dallman MF. Changes in anxiety-related behaviors and hypothalamic-pituitary-adrenal activity in mice lacking the 5-HT-3A receptor. Physiol Behav. 2004;81:545–555.

47.	Qin C, Li J, Tang K. The paraventricular nucleus of the hypothalamus: Development, function, and human diseases. Endocrinology. 2018;159:3458–3472.

48.	Rasiah NP, Loewen SP, Bains JS. Windows into stress: a glimpse at emerging roles for CRHPVN neurons. Physiol Rev. 2023;103:1667–1691.

49.	Daviu N, Füzesi T, Rosenegger DG, Rasiah NP, Sterley T-L, Peringod G, et al. Paraventricular nucleus CRH neurons encode stress controllability and regulate defensive behavior selection. Nat Neurosci. 2020;23:398–410.

50.	Abend R. Understanding anxiety symptoms as aberrant defensive responding along the threat imminence continuum. Neurosci Biobehav Rev. 2023;152:105305.
